# Medical students’ knowledge of the very basic principles of hand hygiene

**DOI:** 10.1186/2047-2994-4-S1-P286

**Published:** 2015-06-16

**Authors:** A Różańska, J Wójkowska-Mach, M Bulanda

**Affiliations:** 1 Chair of Microbiology, Jagiellonian University Medical College, Kraków, Poland

## Introduction

Performing hand hygiene (HH) procedures by the medical staff is a key element in infections prevention. Proper hand hygiene in all situations requiring its use in the Polish health care-settings is still not perfect. In order to improve this situation continuous education of medical personnel using more effective methods should be undertaken. The first stage of such education is the education of medical students of all specialties.

## Objectives

The aim of this study was to examine the state of knowledge of the most basic principles of HH among medical students of Jagiellonian University Medical College.

## Methods

The study was performed by the use of an anonymous questionnaire consisting of 14 questions. The questions concerned the basic data related to the field of study, e.g. type of clinical practice and experience, forms of trainings in the field of HH and HH knowledge.

Total number of 414 completed questionnaires was analyzed. The study was conducted from October to December 2014, among medical students of different specialties. Statistical analysis of the categorical variables was performed using the chi-square test, for the remaining variables - Mann-Whitney and Kruskal-Wallis tests.

## Results

The correct answer to the question about the situation in which HH is necessary was given only by 52.9% of respondents. The correct answer to the question on the choice between hand disinfection and washing, depending on the situation, was given only by 6.5% of respondents. The percentage of correct answers to the questions mentioned above was significantly higher in the group of medical students of the more advanced course. There was no correlation between the duration of clinical practice, or work experience in health care, and knowledge of the principles of HH. As many as one in five respondents declared that clinical practices were not preceded by any training on hospital hygiene.

## Conclusion

The results of this study indicate a basic lack of knowledge in the field of hand hygiene procedures among medical students. Survey results reflect in particular the lack of well-established beliefs of Polish medical staff concerning hand disinfection with alcohol-based hand-rubs. Both, education within the subjects realized in the course of study, and during the clinical training, require improvements, including the implementation of more effective methods.

## Disclosure of interest

None declared.

